# Substitution of free halide ions unlocks responsive photoluminescence switching in manganese-based metal halides

**DOI:** 10.1038/s41377-025-02161-w

**Published:** 2026-02-05

**Authors:** Sisi Li, Kaitong Luo, Yali Zhou, Junhao Wang, Zhen Zhang, Zhao-Qing Liu, Yibo Chen

**Affiliations:** 1 School of Chemistry and Chemical Engineering/Institute of Clean Energy and Materials/Key Laboratory of Guangzhou for Clean Energy and Materials, Guangzhou University, Guangzhou Higher Education Mega Center, Guangzhou, China; 2 Key Laboratory of Organic Integrated Circuits Ministry of Education & Tianjin Key Laboratory of Molecular Optoelectronic Sciences, Department of Chemistry, School of Science, Tianjin University, Tianjin, China

**Keywords:** Optical materials and structures, Optical data storage, Optical sensors

## Abstract

Stimuli-responsive organic-inorganic metal halides hold great promise for emerging information-related applications. In this work, replacing the free halide ion Cl^−^ with Br^−^ in C_5_H_11_N_3_(MnCl_3_·H_2_O)X (where C_5_H_11_N_3_^2+^ represents histamine cation, X represents free halide ions) converts the non-responsive hybrid C_5_H_11_N_3_(MnCl_3_·H_2_O)Cl into a stimuli-responsive C_5_H_11_N_3_(MnCl_3_·H_2_O)Br. The latter exhibits reversible photoluminescence color switching between red and green upon thermal or water exposure. Extensive experimental and theoretical analyses reveal that the responsive property primarily stems from weakened hydrogen bonding surrounding H_2_O molecules after Br^−^ substitution, which facilitates the initial escape of H_2_O molecules under heating. Subsequent structural reorganization and coordination transformation then induce the change in photoluminescence. Furthermore, the fabricated halide/polymer luminescent films are demonstrated to be highly applicable in multiple scenarios, such as planar temperature sensing, thermal stamping, and encryption/decryption. This study highlights the crucial yet often overlooked role of free halide ions in metal halides and offers new insights into their structure–property relationships.

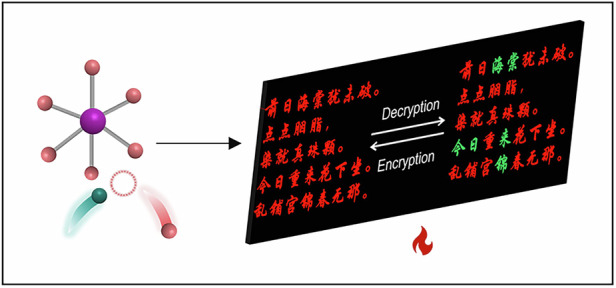

## Introduction

Stimuli-responsive optical materials, which manifest signal output in the spectral forms of absorption, reflection, and luminescence in response to external stimuli^1–3^, have demonstrated significant applications in diverse fields, including but not limited to rewritable paper^4^, remote sensing^5,6^, and information encryption^7–9^. Organic-inorganic metal halides (OIMHs) refer to metal halides with a hybrid structure composed of flexible organic cations and inorganic metal-halide clusters, exhibiting rich crystal structures and diverse luminescent properties^10–16^. Recently, certain OIMHs have been reported to exhibit reversible phase transition and luminescence switching in response to external stimuli such as water, heat, and light, owing to their low formation energy and ionic nature^17,18^. These unique responsive properties of OIMHs endow them as a new generation of smart optical materials with application potential in smart sensing and anti-counterfeiting, thus attracting growing interest^19–21^. However, OIMHs with controllable and reversible stimuli-responsive luminescence remain rare, which greatly limits their practical applications.

Manganese (Mn)-based OIMHs are of particular interest due to their high photoluminescence quantum yields (PLQYs), large Stokes shifts, and stable emission arising from the intrinsic d–d transitions of Mn^2+^ centers^22^. With a 3d^5^ electronic configuration, crystal field-induced d-orbital splitting of Mn^2+^ ions governs their emission wavelength: smaller splitting in weaker crystal fields leads to green or yellow emission, whereas larger splitting in stronger fields shifts the emission to orange or red^23–25^. These characteristics make Mn-based OIMHs ideal platforms for exploring multicolor stimuli-responsive luminescent materials. In recent years, an increasing number of Mn-based OIMHs showing solvent-, pressure-, thermal-, and photo-responsive luminescence properties have been reported^26–31^. For example, a photoluminescence shifting from green to red is observed in [PP14]_2_[MnBr_4_] ([PP14]^+^ = N-butyl-N-methylpiperidinium) over a pressure range of 1 atm to 12.5 GPa^28^. This impressive piezochromism in its luminescence can be ascribed to the increased crystal field splitting energy under high pressure. In another case, a zero-dimensional (0D) green-emitting Mn-based OIMH, (DPPE)_2_MnBr_4_·H_2_O (DPPE = 4,4-Difluoropiperidine), has been reported to transform into a red-emitting hybrid upon treatment of dichloromethane, due to solvent-induced coordination configuration transformation^29^. Despite these advances, the underlying relationships between the crystal structure and the responsive property in Mn-based OIMHs are still not well understood, posing challenges to the rational design of response-tunable systems.

Free halide ions refer to halide ions present in the OIMHs lattice serving charge-balancing functions but not directly coordinated to metal centers^32,33^. In this work, we synthesized two single-crystalline OIMHs, C_5_H_11_N_3_(MnCl_3_·H_2_O)Cl containing free Cl^−^ and C_5_H_11_N_3_(MnCl_3_·H_2_O)Br containing free Br^−^. We demonstrate that C_5_H_11_N_3_(MnCl_3_·H_2_O)Br displays reversible photoluminescence color switching between green and red upon thermal and moisture exposure, while its Cl⁻ counterpart shows no response under the same conditions. A distinct structure-property relationship between the free halide ions and the responsive photoluminescence is derived by detailed experimental and theoretical analyses. Following Br⁻ substitution, the weakened hydrogen bonding surrounding H_2_O molecules facilitates the escape of H_2_O molecules and enables structural rearrangement upon stimuli exposure, leading to coordination geometry alteration and the photoluminescence color change. Further, the potential applications of the C_5_H_11_N_3_(MnCl_3_·H_2_O)Br halide in planar temperature sensing, thermal stamping, and information encryption are showcased.

## Results

### Crystal structures and optical properties

**Fig. 1:**
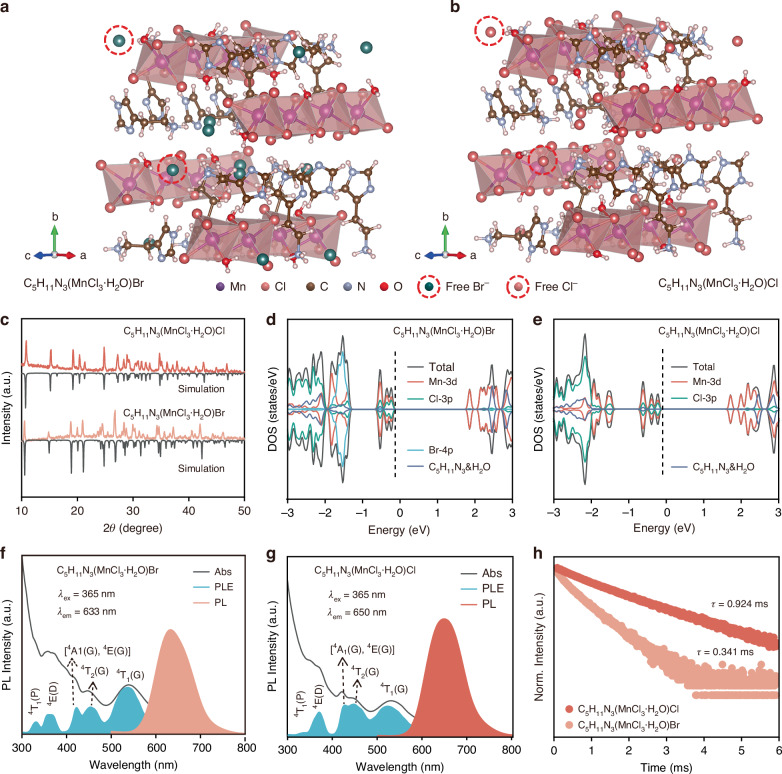
Structural and optical characterizations. **a**, **b** Crystal structure diagrams of C_5_H_11_N_3_(MnCl_3_·H_2_O)Br (**a**) and C_5_H_11_N_3_(MnCl_3_·H_2_O)Cl (**b**), with the free halide ions highlighted in red circles. **c** Simulated PXRD from single crystal diffraction data and experimental PXRD of powder samples. **d**, **e** Calculated density of states. **f**, **g** PL, PLE, and absorption spectra. **h** PL decay curves

Two new Mn-based hybrid halides crystals, C_5_H_11_N_3_(MnCl_3_·H_2_O)X (X = Cl^−^, or Br^−^), were successfully synthesized via an evaporative crystallization method (Please find the details in the Materials and methods section). The crystal structures of C_5_H_11_N_3_(MnCl_3_·H_2_O)Br and C_5_H_11_N_3_(MnCl_3_·H_2_O)Cl were determined via single crystal X-ray diffraction (SCXRD). The two metal halides both crystallize in the tetragonal space group $P\bar{4}{2}_{1}c$. As illustrated in Fig. 1a, b, each Mn atom is coordinated by five Cl atoms and one H_2_O molecule, forming a [MnCl_3_·H_2_O]^−^ octahedron. The edge-sharing octahedral units are interconnected along the *c*-axis to form a 1D arrangement, which is separated by the C_5_H_11_N_3_^2+^ organic cations. Notably, free halide ions (Cl⁻ or Br⁻, marked by red circles) are found in the structures to maintain the charge balance. The detailed lattice parameters are shown in Table S1. The experimental powder X-ray diffraction patterns (PXRD) of the two Mn-based metal halides powders are in close agreement with their simulated counterparts (Fig. 1c), confirming the successful synthesis of the products.

High-resolution X-ray photoelectron spectroscopy (XPS) spectra (Fig. S1) demonstrate the localized electronic structure modification after substituting free Cl^−^ with Br^−^. The calculated density of states (DOS) shows that the valence band maximum (VBM) of the two metal halides is mainly composed of Mn-3d and Cl-3p orbitals, and the conduction band minimum (CBM) is mainly contributed by Mn-3d (Fig. 1d, e). The DOS profiles of the two crystals are quite similar, as well as the calculated direct bandgaps of 2.090 eV for C_5_H_11_N_3_(MnCl_3_·H_2_O)Br and 1.941 eV for C_5_H_11_N_3_(MnCl_3_·H_2_O)Cl. It means that the band structures of the two crystals are primarily contributed by the Mn-Cl polyhedrons, with negligible involvement of the free halide ions. The experimental bandgap values were derived by fitting the absorption spectra according to Tauc’s equation (Fig. S2). The values are 3.94 and 3.99 eV for C_5_H_11_N_3_(MnCl_3_·H_2_O)Cl and C_5_H_11_N_3_(MnCl_3_·H_2_O)Br, respectively. The calculated bandgap values fall short of the experimental ones, mainly due to the intrinsic limitations inherent in the Perdew−Burke−Ernzerhof (PBE) approximation when it comes to accurately estimating bandgaps. Notably, the similarity in bandgap values between the two crystals aligns with the calculated results, confirming that the substitution of free halide ions does not alter the band structure.

Both C_5_H_11_N_3_(MnCl_3_·H_2_O)Cl and C_5_H_11_N_3_(MnCl_3_·H_2_O)Br crystals are colorless and transparent crystals under natural light while emitting bright red light when exposed to 365 nm irradiation (Fig. S3). The photoluminescence excitation (PLE), and photoluminescence (PL) spectra of these Mn-based halides are illustrated in Fig. 1f, g. The PLE spectrum of C_5_H_11_N_3_(MnCl_3_·H_2_O)Br consists of several bands, with peaks centered at approximately 332, 366, 422, 455, and 536 nm. These excitation bands correspond to the electronic transitions from the ^6^A_1_ ground state of Mn^2+^ to the ^4^T_1_(P), ^4^E(D), [^4^A_1_(G), ^4^E(G)], ^4^T_2_(G), and ^4^T_1_(G) excited states, respectively^34,35^. The PLE spectra of C_5_H_11_N_3_(MnCl_3_·H_2_O)Cl exhibits similar excitation bands. However, their central wavelengths show slight shifts, with peaks located at around 337, 371, 428, 448, and 523 nm, respectively. The absorption spectra are matchable with the PLE spectra. The red emission of the Mn-based metal halides originates from the ^4^T_1_ → ^6^A_1_ electron transition of Mn^2+^ ^36^. Upon 365 nm excitation, both of the hybrids show broad PL bands, with a full width at half maximum (FWHM) of ~90 nm, consisting with the previous reports^37,38^. The C_5_H_11_N_3_(MnCl_3_·H_2_O)Br shows red emission located at 633 nm while C_5_H_11_N_3_(MnCl_3_·H_2_O)Cl exhibits an emission peak at 650 nm. The shift in luminescence color is visually shown in the CIE chromaticity diagram (Fig. S4), with the chromaticity coordinates (0.6538, 0.3294) for C_5_H_11_N_3_(MnCl_3_·H_2_O)Cl and (0.6202, 0.3425) for C_5_H_11_N_3_(MnCl_3_·H_2_O)Br.

Why does the substitution of free halide ions lead to a 17 nm blueshift in the emission? This phenomenon can be rationalized by considering the differences in crystal field strength. The crystal field strength (*Dq*) can be calculated as follows^39^:

1 $${Dq}=z{{\rm{e}}}^{2}{r}^{4}/6{R}^{5}$$

where *z* denotes the charge of the coordinated ion; *e* is the electronic charge; *r* denotes the radius of the *d* wave function; and *R* is the average distance between the central atom and the ligand. The results as detailed in Table S2 indicate that the average distance (*R*) between the Mn^2+^ center and the ligand in C_5_H_11_N_3_(MnCl_3_·H_2_O)Br (2.5059 Å) is more pronounced compared to that in C_5_H_11_N_3_(MnCl_3_·H_2_O)Cl (2.4807 Å). This indicates that the substituted free halide ions substantially alter the coordination environment around the Mn^2+^ center, even though they do not directly coordinate with Mn^2+^. The larger value of *R* in C_5_H_11_N_3_(MnCl_3_·H_2_O)Br then leads to a decrease in the crystal field strength, which consequently diminishes the splitting of d-orbital energy levels, thus elevating the excited energy level of Mn^2+^ and inducing a blueshift in the emission.

PL decay curves of C_5_H_11_N_3_(MnCl_3_·H_2_O)Cl and C_5_H_11_N_3_(MnCl_3_·H_2_O)Br are shown in Fig. 1h, and the decay lifetimes of the two are determined to be 0.924 and 0.341 ms (Table S3). The results indicate that the non-radiative luminescence loss increases with the substitution of free Cl⁻ by free Br⁻, which is consist with the PLQY values of 65.4% and 26.4% for C_5_H_11_N_3_(MnCl_3_·H_2_O)Cl and C_5_H_11_N_3_(MnCl_3_·H_2_O)Br (Fig. S5). The loss can be explained by the larger octahedral distortion index (DI) of 0.0303 in C_5_H_11_N_3_(MnCl_3_·H_2_O)Br, whereas the DI value in C_5_H_11_N_3_(MnCl_3_·H_2_O)Cl is 0.0267 (Table S2). Significant structural distortion of the inorganic units can introduce more defects and result in a lower PLQY^40,41^.

### Responsive photoluminescence switching

**Fig. 2:**
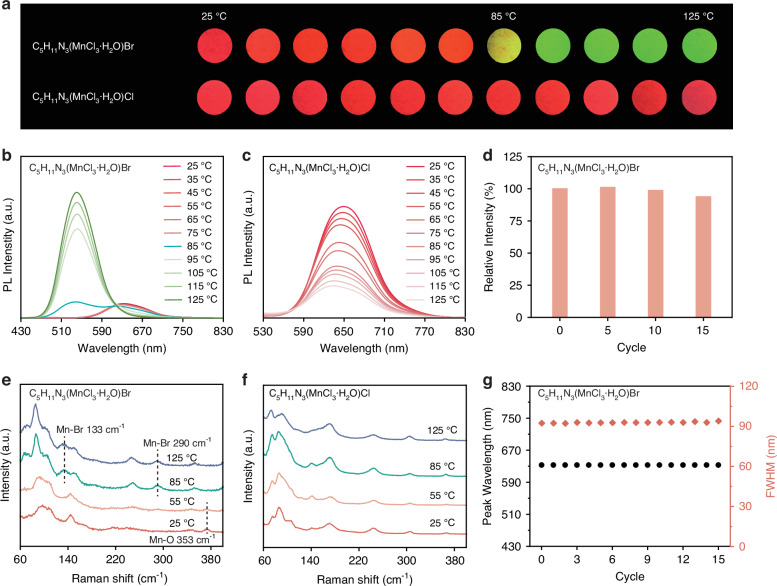
**Reversible response properties under heating/cooling conditions.** **a** Photographs showing the luminescence variations of C_5_H_11_N_3_(MnCl_3_·H_2_O)Br and C_5_H_11_N_3_(MnCl_3_·H_2_O)Cl at different temperatures. **b**, **c** Temperature-dependent PL spectra. **e**, **f** Temperature-dependent Raman spectra. **d** PL intensity of C_5_H_11_N_3_(MnCl_3_·H_2_O)Br within 15 cycles. **g** Peak position and FWHM of C_5_H_11_N_3_(MnCl_3_·H_2_O)Br within 15 cycles

The substitution of Br^−^ enables a responsive PL switching, that the PL color of C_5_H_11_N_3_(MnCl_3_·H_2_O)Br changes from red to green when heated from 25 to 125 °C (Fig. 2a). As shown in Fig. 2b, a distinct dual-band emission (cyan line) with the peaks located at 633 and 537 nm is identified at 85 °C. In contrast, C_5_H_11_N_3_(MnCl_3_·H_2_O)Cl maintains stable red emission across the entire temperature range from 25 to 125 °C (Fig. 2c). More detailed spectra are shown in Fig. S6 to confirm the difference between the two samples. The conversion of PL color from red to green suggests that the Mn-centered polyhedron deforms from octahedral to tetrahedral configuration^42^. PXRD patterns of C_5_H_11_N_3_(MnCl_3_·H_2_O)Br recorded at different temperatures reveal phase evolution during the heating process, indicating that the change in PL color is likely attributed to a crystal-to-crystal phase transition (Fig. S7). The well-defined diffraction peaks demonstrate that no amorphous phase was generated during this process. The PL profile of C_5_H_11_N_3_(MnCl_3_·H_2_O)Br fully recovers after an “initial→heating→cooling” cycle (Fig. S8), and the PXRD patterns of the sample after a heating-cooling treatment are in good agreement with those of the initial sample (Fig. S9). Figure 2d, g displays the changes in various PL parameters of C_5_H_11_N_3_(MnCl_3_·H_2_O)Br over 15 cycles. Only a 6% decrease in PL intensity is observed, and the peak position and FWHM of the PL spectra remain stable. The above results confirm that the thermally responsive PL switching property of C_5_H_11_N_3_(MnCl_3_·H_2_O)Br is highly reversible.

Temperature-dependent Raman characterization was conducted to reveal the bonding changes in C_5_H_11_N_3_(MnCl_3_·H_2_O)Br at high temperatures (Fig. 2e). In 25–55 °C, the Raman spectra of C_5_H_11_N_3_(MnCl_3_·H_2_O)Br show distinct stretching modes at ~353 cm^−1^, which are vibrations mostly from Mn-O bonds^43,44^. Upon increasing the temperature above 85 °C, new Raman peaks emerge at 133 and 290 cm^−1^, which are attributed to the Mn-Br vibrational modes^28,45^. Concurrently, the Mn-O vibrational peak at 353 cm^−1^ disappears, corresponding to the loss of coordinated H_2_O molecules. On the other hand, the Raman spectra of C_5_H_11_N_3_(MnCl_3_·H_2_O)Cl show negligible changes during the heating course (Fig. 2f). The Raman spectra provide extra evidence that C_5_H_11_N_3_(MnCl_3_·H_2_O)Br undergoes a crystal-to-crystal phase transition at 85 °C, also reveal that the removal of coordinated H_2_O molecules and the formation of Mn-Br bonds are key steps in the structural transformation process.

### Structural evolution mechanism

**Fig. 3:**
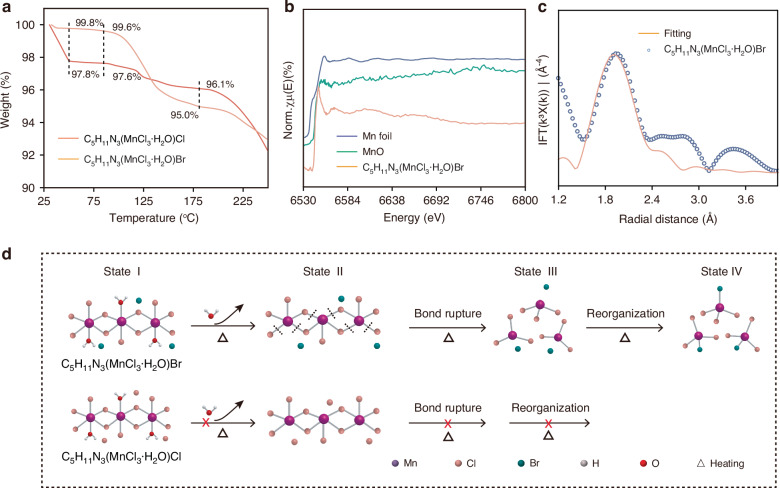
**Structural evolution steps.** **a** TGA curves of C_5_H_11_N_3_(MnCl_3_·H_2_O)Br and C_5_H_11_N_3_(MnCl_3_·H_2_O)Cl. **b** XANES spectrum of C_5_H_11_N_3_(MnCl_3_·H_2_O)Br. **c** Fourier-transformed EXAFS spectrum of C_5_H_11_N_3_(MnCl_3_·H_2_O)Br in R space with fitting result. **d** Proposed structural evolution process for C_5_H_11_N_3_(MnCl_3_·H_2_O)Br and C_5_H_11_N_3_(MnCl_3_·H_2_O)Cl under heating

To further clarify the structural evolution upon heating, thermogravimetric analysis (TGA), and X-ray absorption near-edge structure (XANES) measurements were performed. TGA results of C_5_H_11_N_3_(MnCl_3_·H_2_O)Br and C_5_H_11_N_3_(MnCl_3_·H_2_O)Cl are shown in Fig. 3a, and the corresponding differential scanning calorimetry (DSC) results are shown in Fig. S10. The C_5_H_11_N_3_(MnCl_3_·H_2_O)Br experiences a substantial weight loss of 4.6% at 85–180 °C, closely matching the theoretical mass fraction of coordinated H_2_O molecules (4.8%) derived from the chemical formula. Moreover, a significant endothermic peak is observed in the DSC curve within the temperature range of 85–180 °C. These results confirm that the structural evolution initiates with the escape of the coordinated H_2_O molecules. However, C_5_H_11_N_3_(MnCl_3_·H_2_O)Cl does not exhibit a comparable loss of coordinated H_2_O molecules, which theoretically accounts for 5.5% of the total weight. It only shows a minor weight loss of 2.2% below 50 °C, which can be attributed to the release of adsorbed solvent molecules. The XANES and extended X-ray absorption fine structure (EXAFS) spectra were collected after the C_5_H_11_N_3_(MnCl_3_·H_2_O)Br was heated to 100 °C and maintained for 10 min, aiming to define the structural features of the sample post-heating. As illustrated in Fig. 3b, the Mn K-edge XANES spectrum is in good agreement with that of the standard MnO, thereby confirming that the valence state of Mn is 2. Figure 3c shows the Fourier-transformed EXAFS spectrum in R-space and the fitting line, which reveal the bond lengths and coordination number information about Mn^2+^ (Table S4). The coordination number of the green-emitting post-heating product is set to be 4, and the coordination ratio of Cl to Br is fitted to be approximately 3:1. Consequently, the chemical formula of the green-emitting product can be expressed as C_5_H_11_N_3_MnCl_3_Br.

Based on the above results, we propose the chemical reaction equation for the thermal-activated structural transition of C_5_H_11_N_3_(MnCl_3_·H_2_O)Br as:

2 $${C}_{5}{H}_{11}{N}_{3}\left({MnC}{l}_{3}\cdot {H}_{2}O\right){Br}\frac{{heating}}{\bar{{cooling}}}{C}_{5}{H}_{11}{N}_{3}{MnC}{l}_{3}{Br}+{H}_{2}O$$

The proposed structural evolution steps are shown in Fig. 3d. The original state of C_5_H_11_N_3_(MnCl_3_·H_2_O)Br is defined as State I. The thermal treatment induces the escape of coordinated H_2_O molecules, resulting in a decrease in the number of coordinated atoms around Mn^2+^ (State II). In addition, heating induces energy perturbation within the crystal, which promotes the splitting of the 1D Mn-Cl polyhedron chain into separated triangular configurations (State III). Since the triangular state is vulnerable, it is inclined to coordinate with the free Br^−^ to form a [MnCl_3_Br]^2−^ tetrahedron (State IV). However, C_5_H_11_N_3_(MnCl_3_·H_2_O)Cl fails to lose its coordinated H_2_O upon heating (as indicated by TGA), which blocks the subsequent bond-rupture and structural reorganization steps. Thus, no changes in the structure or luminescent properties can be induced under thermal stimulation in C_5_H_11_N_3_(MnCl_3_·H_2_O)Cl. A control experiment was conducted to verify the structural evolution process. After the C_5_H_11_N_3_(MnCl_3_·H_2_O)Br powders were carefully sealed, the temperature-dependent PL spectra of the sealed sample were recorded. In chemical reaction Eq. (2), the loss of H_2_O is critical for the chemical reaction to proceed in the forward direction. If the H_2_O molecules cannot be removed, the reaction will not occur. Just as expected, the sealed sample in which the H_2_O molecules cannot escape, persistently maintains red emission during the heating process or even being kept at 90 °C for 60 min (Fig. S11). Thus, the structural evolution process we proposed is consistent with the experimental observations and is therefore reasonable.

**Fig. 4:**
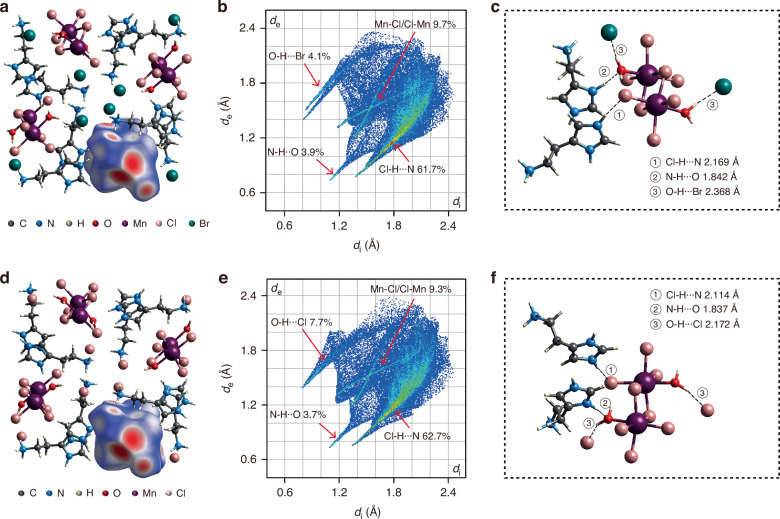
**Hirshfeld surface analysis.** **a**–**c** Hirshfeld surface (**a**), 2D fingerprint plots (**b**), and hydrogen bonds scheme (**c**) in C_5_H_11_N_3_(MnCl_3_·H_2_O)Br. **d**–**f** Hirshfeld surface (**d**), 2D fingerprint plots (**e**), and hydrogen bonds scheme (**f**) in C_5_H_11_N_3_(MnCl_3_·H_2_O)Cl

Hirshfeld surface calculations were performed to investigate the weak interactions between the Mn-centered octahedron and the surrounding moieties^46^. The calculated Hirshfeld surfaces for C_5_H_11_N_3_(MnCl_3_·H_2_O)Br and C_5_H_11_N_3_(MnCl_3_·H_2_O)Cl are presented in Fig. 4a, d, respectively. Red areas on the surface indicate interactions stronger than van der Waals forces. The contributions of different interactions to the overall surface are depicted in the 2D fingerprint plots (Fig. 4b, e), with the corresponding hydrogen bonds are schematically shown in Fig. 4c, f. The Cl-H···N hydrogen bonding (denoted as ①) plays a major role in the interactions between the octahedron and the organic parts. The Mn-Cl/Cl-Mn interactions reflect the mutual interactions of the octahedrons in the 1D chains. Following in terms of contribution are the interactions around the H_2_O molecules, including N-H···O hydrogen bonds (denoted as ②) and O-H···Cl(Br) (denoted as ③, where Cl/Br is a free halide ion). All hydrogen bond lengths in C_5_H_11_N_3_(MnCl_3_·H_2_O)Br are longer than these in C_5_H_11_N_3_(MnCl_3_·H_2_O)Cl, indicating weaker bonding strength. Notably, the O-H···Br bonding in C_5_H_11_N_3_(MnCl_3_·H_2_O)Br not only has a longer bond length but also makes a smaller contribution than the counterpart O-H···Cl bonding in C_5_H_11_N_3_(MnCl_3_·H_2_O)Cl. Combined with the above structural evolution steps, we infer that it is the weakened hydrogen bonding interactions around the H_2_O molecules after Br^−^ substitution that facilitate the escape of H_2_O molecules under heating. The departure of these H_2_O molecules in C_5_H_11_N_3_(MnCl_3_·H_2_O)Br then triggers a structural reorganization, which alters the coordination geometry of Mn^2+^ from a six-coordinated octahedron to a four-coordinated tetrahedron. The weak crystal filed in a tetrahedron leads to a significant change in its luminescence color.

### Diverse application demos

**Fig. 5:**
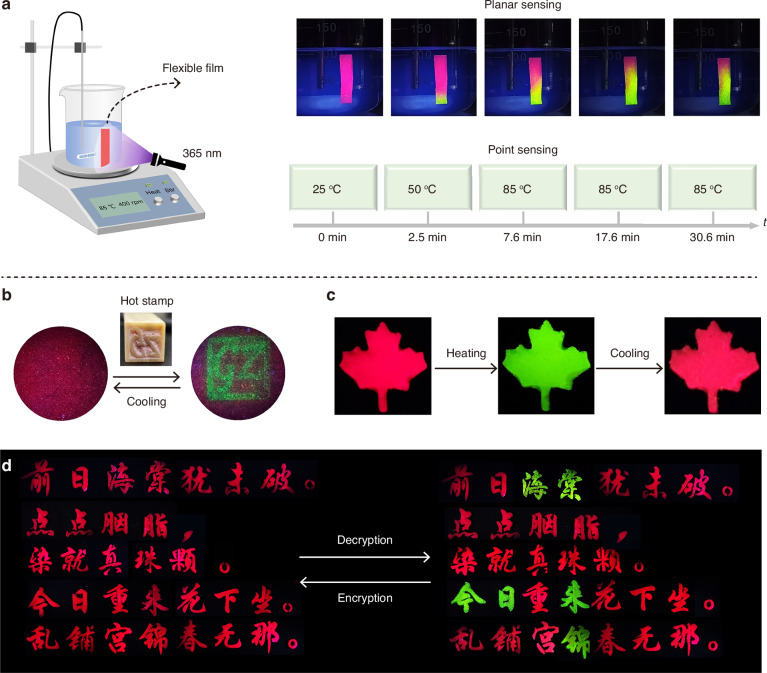
**Diverse application demos.** **a** Testing device set and the planar sensing results for visualized temperature monitoring. **b** Thermal stamping on a C_5_H_11_N_3_(MnCl_3_·H_2_O)Br/PDMS film using a pre-heated stone stamp (15 × 15 mm^2^) with the “GZ” protrusive symbol. **c** Photographs of a “maple leaf” luminescent anti-counterfeiting label made of C_5_H_11_N_3_(MnCl_3_·H_2_O)Br. **d** Hiding the secret message “Begonia is coming to Jin today” in a Chinese ci poem made of C_5_H_11_N_3_(MnCl_3_·H_2_O)Br/PDMS and C_5_H_11_N_3_(MnCl_3_·H_2_O)Cl/PDMS composites

Planar temperature sensing or 2D thermal imaging can provide richer spatial information, such as thermal transportation characteristics and temperature dynamic distributions, than traditional point temperature sensing^47,48^. Here we fabricated a flexible film (25 × 5 × 0.8 mm^3^) by mixing C_5_H_11_N_3_(MnCl_3_·H_2_O)Br with polydimethylsiloxane (PDMS), denoted as C_5_H_11_N_3_(MnCl_3_·H_2_O)Br/PDMS (Figs. S12 and S13). This film was employed to spatially monitor the temperature distribution across the surface of a beaker containing 30 mL of silicone oil. The heating temperature was set to 85 °C. As illustrated in Fig. 5a, the heating table registers a temperature of 50 °C after 2.5 min of heating. However, the film shows green emission at the bottom, which means that the bath bottom has reached at least 85 °C. Upon continued heating for an additional 5 min, the green emission area in the film increases significantly. After being maintained at this temperature for 23 min, the green light area tends to stabilize, indicating that the silicone oil has reached a steady temperature state. The green light does not cover the entire film, which reflects the inhomogeneity of the temperature distribution inside the silicone oil. In contrast, the heating table shows limited information during the heating process. Although it gives the same information “85 °C” at 7.6, 17.6, and 30.6 min, the real case is more complicated. These findings verify that the C_5_H_11_N_3_(MnCl_3_·H_2_O)Br/PDMS film is capable of visually reflecting the real-time temperature distribution. The application of the C_5_H_11_N_3_(MnCl_3_·H_2_O)Br/PDMS film for thermal stamping is demonstrated in Fig. 5b. A stone stamp (15 × 15 mm^2^) with a “GZ” protrusion was preheated to 150 °C. Then, it was pressed onto the film surface for 10 s to print a green-lighting “GZ” pattern. After approximately 10 min, the film can restore to its original red luminescence by absorbing the ambient moisture. This straightforward hot stamping technique enables thermal-stimulated writing and tracking in a simple and reusable manner.

The hybrid can also be applied in the field of anti-counterfeiting and information security. The C_5_H_11_N_3_(MnCl_3_·H_2_O)Br powders were filled into the “maple leaf” pattern, where it emits red light under 365 nm light. Upon thermal treatment at 90 °C, the red emission shifts to green and reverts to red after cooling (Fig. 5c). This reversible PL thermochromic behavior enables C_5_H_11_N_3_(MnCl_3_·H_2_O)Br to be used as a dynamic anti-counterfeiting label. Figure 5d illustrates the information encryption demo utilizing C_5_H_11_N_3_(MnCl_3_·H_2_O)Br/PDMS and C_5_H_11_N_3_(MnCl_3_·H_2_O)Cl/PDMS as raw materials. An excerpt from a Chinese ci poem was mask-printed onto a glass substrate using the two materials. Initially, all the characters emit red emission under 365 nm illumination. Then, heating was employed to reveal the encrypted text. The characters made of C_5_H_11_N_3_(MnCl_3_·H_2_O)Br/PDMS changed from red to green emission upon thermal stimulation, whereas those made of C_5_H_11_N_3_(MnCl_3_·H_2_O)Cl/PDMS retain their red emission, thereby decrypting the secret message “Begonia is coming to Jin today”. Subsequent cooling fully restored the initial pattern and re-established the encryption. In a word, multiple application scenarios including planar temperature monitoring, thermal stamping, and information security have been established, demonstrating the application potential of the responsive Mn-based hybrid halide.

## Discussion

In summary, two organic-inorganic metal halide single crystals, C_5_H_11_N_3_(MnCl_3_·H_2_O)Br and C_5_H_11_N_3_(MnCl_3_·H_2_O)Cl, were successfully synthesized, and a distinct structure-property relationship between the free halide ions and the responsive PL has been derived. Despite sharing the same crystal space group ($P\bar{4}{2}_{1}c$), these two hybrids exhibit different PL response behaviors. The introduction of free Br⁻ endows C_5_H_11_N_3_(MnCl_3_·H_2_O)Br with the reversible PL color switching between red and green under thermal/water stimulation, a property that is absent in C_5_H_11_N_3_(MnCl_3_·H_2_O)Cl. This PL switching shows excellent reversibility, maintaining stable performance over 15 cycles with negligible shifts in PL wavelength and minimal loss of intensity. Detailed temperature-dependent characterizations and theoretical results reveal that it is the weaker hydrogen bonding surrounding H_2_O molecules in C_5_H_11_N_3_(MnCl_3_·H_2_O)Br drives loss of the coordinated H_2_O molecules upon heating. Subsequent structural reorganization induces the change in the coordination configuration of the Mn center, which accounts for the observed PL switching. Further, the application capacity of the responsive luminescent Mn-based halide has been demonstrated in multiple scenarios, including planar temperature sensing, thermal stamping, and information encryption. This work elucidates the role of free halide ions in modulating the responsive performance, opening a new pathway for the development of smart metal halides and their applications in cutting-edge information-related fields.

## Materials and methods

### Chemicals

All chemical materials were purchased and used directly without further purification. Histamine dihydrochloride (C_5_H_9_N_3_·2HCl, B.R., 98%), manganese chloride tetrahydrate (MnCl_2_·4H_2_O, B.R., 99.99%), and manganese bromide tetrahydrate (MnBr_2_·4H_2_O, B.R., 98%) were obtained from Shanghai Aladdin Reagent. Isopropanol (C_3_H_8_O, A.R.) was obtained from Shanghai Macklin Reagent. Polydimethylsiloxane (PDMS) and the curing agent were purchased from Dow Corning Reagent. Hydrochloric acid (HCl, 37%) was purchased from Guangzhou Chemical Reagent.

### Synthesis of single crystals

The single-crystal samples were prepared with a slow solvent evaporation method. C_5_H_9_N_3_·2HCl (1.0 mmol) and MnCl_2_·4H_2_O (1.0 mmol) were dissolved in 2 mL of pure water, and then 2 mmol of 10% HCl solution was added to the solution. The addition of HCl solution is to maintain a high Cl^−^ concentration, which favors growth of the crystals. The solution was then slowly evaporated at 40 °C to obtain C_5_H_11_N_3_(MnCl_3_·H_2_O)Cl single crystal. For the preparation of C_5_H_11_N_3_(MnCl_3_·H_2_O)Br single crystal, C_5_H_9_N_3_·2HCl (1.0 mmol) and MnBr_2_·4H_2_O (1.0 mmol) were dissolved in 2 mL of pure water. Subsequently, 150 μL of the above solution was injected into 10 mL of isopropanol. After well shaken, the solution was then slowly evaporated at 40 °C to obtain C_5_H_11_N_3_(MnCl_3_·H_2_O)Br single crystal. Compared with the previous report^26^, isopropanol is used as a poor solvent in our work to induce the crystallization. The X-ray crystallographic data of the two crystals has been deposited at the Cambridge Crystallographic Data Centre (CCDC), under deposition numbers 2457548 (for C_5_H_11_N_3_(MnCl_3_·H_2_O)Cl) and 2457549 (for C_5_H_11_N_3_(MnCl_3_·H_2_O)Br).

### Synthesis of powder samples

C_5_H_9_N_3_·2HCl (1.0 mmol) and MnCl_2_·4H_2_O (1.5 mmol) were dissolved in 2 mL of pure water. The above solution was then heated at 120 °C and evaporated under stirring until a precipitate appeared. Subsequently, the precipitate was separated and washed with isopropanol. After been dried under vacuum at 70 °C, the final C_5_H_11_N_3_(MnCl_3_·H_2_O)Cl powder product was obtained. For the preparation of C_5_H_11_N_3_(MnCl_3_·H_2_O)Br powder, C_5_H_9_N_3_·2HCl (1.0 mmol) and MnBr_2_·4H_2_O (1.0 mmol) were dissolved in 2 mL of pure water. The above solution was then heated at 50 °C and slowly evaporated until a precipitate appeared. The precipitate was separated, washed, and further dried under vacuum at 50 °C to obtain the final product.

### Preparation of C_5_H_11_N_3_(MnCl_3_·H_2_O)Br/PDMS

The mixture of PDMS prepolymer and curing agent (prepolymer: curing agent ≈ 9:1) were weighed to be 1.0 g, then 0.03 g of C_5_H_11_N_3_(MnCl_3_·H_2_O)Br powder sample was added. After homogeneously mixed, the mixture was heated at 70 °C for 1 h to cure the polymer. Please find the characterization details in the SI file.

## Supplementary information

Supplementary Information File

## Data Availability

All relevant data that support the findings of this work are available from the corresponding author on reasonable request.
