# Supplementary material for: Substitution of free halide ions unlocks responsive photoluminescence switching in manganese-based metal halides
Source: Light Sci Appl. 2026 Feb 5;15:105. doi: 10.1038/s41377-025-02161-w (PMC12873375; doi:10.1038/s41377-025-02161-w)
Supplement: Supplementary file 1 — Supplementary Information File [file 41377_2025_2161_MOESM1_ESM.docx]

*Supplementary Information for*

**Substitution of free halide ions unlocks** **responsive photoluminescence switching in manganese-based metal halides**

*Sisi Li^a^, Kaitong Luo^a^,Yali Zhou^a^, Junhao Wang^b^,* *Zhen Zhang^b^, Zhao-Qing Liu^a^, Yibo Chen^*a^*

*^a^School of Chemistry and Chemical Engineering/Institute of Clean Energy and Materials/Key Laboratory of Guangzhou for Clean Energy and Materials, Guangzhou University, Guangzhou Higher Education Mega Center No. 230 Wai Huan Xi Road, 510006, P. R. China*

*^b^Key Laboratory of Organic Integrated Circuits Ministry of Education & Tianjin Key Laboratory of Molecular Optoelectronic Sciences, Department of Chemistry, School of Science, Tianjin University, Tianjin 300072, P. R. China*

**Corresponding author: Yibo Chen, E-mail:* [*chenyibo@gzhu.edu.cn*](mailto:chenyibo@gzhu.edu.cn)*.*

**1. Characterization Details**

Single crystal X-ray diffraction data (SCXRD) was collected at 100 K by Rigaku XtaLAB of Synergy, Japan. The crystal structures were refined by the Olex2 software. Powder X-ray diffraction (PXRD) patterns were measured by a PANalytical PW3040/60 X-ray powder diffractometer. A X-ray photoelectron spectroscopy (XPS, Thermo Scientific-ESCALAB Xi+, USA) was used to investigate the electronic state of the samples. The UV-vis absorption spectra were collected using an American PerkinElmer Lambda950 spectrophotometer. The photoluminescence excitation (PLE) spectra, photoluminescence quantum yields (PLQY), and lifetime spectra were measured using an Edinburgh FLS 1000 fluorescence spectrometer with a 450 W xenon lamp as light source. The responsive PL spectra were recorded on a fiber optic spectrometer (Ocean Optics USB 2000+) coupled with a 365 nm UV lamp and a heating platform. Note that the above response measurement was performed on a 10 × 10 mm^2^ film assembled by approximately 0.043 g metal halide powder samples. To evaluate the impact of H_2_O escape during the phase transition process, halide samples were sealed between two glass slides as control samples. The sample powders were first sandwiched between the two slides, and the gaps in the slide pairs were then sealed with UV adhesive, which cures in ~10 s under UV irradiation. Thermogravimetric analysis (TGA)-differential scanning calorimetry (DSC) results were collected by a Hitachi-STA200 instrument. The powder was heated from 25 to 250 °C at a heating rate of 10 ℃/min in an air environment. Temperature-dependent Raman spectra were recorded using a Raman spectrometer (LabRAM HR Evolution) with a 785 nm laser in 25–125 °C. X-ray absorption near-edge structure (XANES) spectroscopy and extended X-ray absorption fine structure (EXAFS) spectra were acquired using a X-ray absorption fine structure (XAFS) spectrometer (Table XAFS-500-A) at 100 °C using boron nitride powder to dilute the samples.

Information on the type and number of hydrogen bonds was obtained using CrystalExplorer 2.1 software. First-principle calculations were performed by the density functional theory (DFT) using the Vienna Ab-initio Simulation Package (VASP) package^1^. The generalized gradient approximation (GGA) with the Perdew−Burke−Ernzerhof (PBE) functional were used to describe the electronic exchange and correlation effects^2–4^. Uniform G-centered k-points meshes with a resolution of 2π × 0.05 Å^−1^ and Methfessel-Paxton electronic smearing were adopted for the integration in the Brillouin zone for geometric optimization. The simulation was run with a cutoff energy of 500 eV throughout the computations. The geometry optimization was considered convergent when the electronic energy and Hellmann-Feynman forces convergence criterion was smaller than 10^−5^ eV and 0.03 eV Å^−1^, respectively.

**2. Supplementary figures**


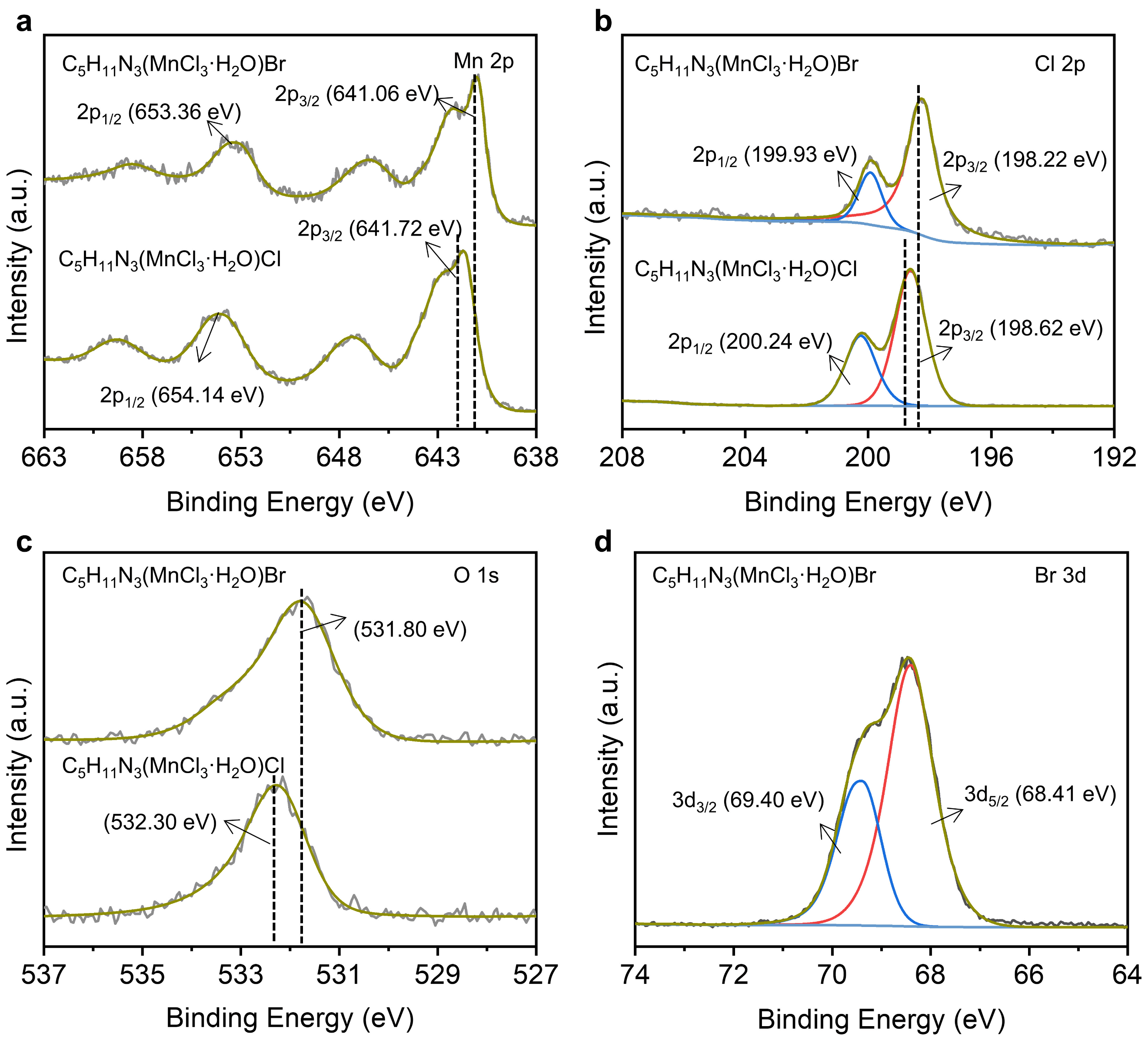


**Fig. S1 a–d** High-resolution XPS spectra and fitting results of Mn 2p **(a)**, Cl 2p **(b)**, O 1s **(c)**, and Br 3d **(d)** in C_5_H_11_N_3_(MnCl_3_·H_2_O)Cl and C_5_H_11_N_3_(MnCl_3_·H_2_O)Br.

**Note**: The XPS results demonstrate that the localized electronic structure has been modified by substituting free Cl^−^ with Br^−^. The Mn 2p spectrum of C_5_H_11_N_3_(MnCl_3_·H_2_O)Cl shows 2p_3/2_ and 2p_1/2_ peaks at the binding energies of 641.72 and 654.14 eV, respectively^5^, while the peaks of Mn 2p_3/2_ and Mn 2p_1/2_ are at 641.06 and 653.36 eV in C_5_H_11_N_3_(MnCl_3_·H_2_O)Br. Compared with those of C_5_H_11_N_3_(MnCl_3_·H_2_O)Cl, the peaks of Mn 2p_3/2_ and 2p_1/2_ shift to lower binding energy values in C_5_H_11_N_3_(MnCl_3_·H_2_O)Br, indicating an increase in the electron cloud density around Mn atom in C_5_H_11_N_3_(MnCl_3_·H_2_O)Br. Similarly, the binding energy values of Cl 2p_3/2_ (198.22 eV) and Cl 2p_1/2_ (199.93 eV) in Cl 2p spectra of C_5_H_11_N_3_(MnCl_3_·H_2_O)Br shift to lower ones compared to Cl 2p_3/2_ (198.62 eV) and Cl 2p_1/2_ (200.24 eV) in C_5_H_11_N_3_(MnCl_3_·H_2_O)Cl^6^. The binding energy of O 1s (531.8 eV) in C_5_H_11_N_3_(MnCl_3_·H_2_O)Br shifts to a lower value by 0.5 eV with respect to that in C_5_H_11_N_3_(MnCl_3_·H_2_O)Cl^5^. Distinct Br 3d peaks at 68.41 eV (3d_3/2_) and 69.40 eV (3d_5/2_) appear in C_5_H_11_N_3_(MnCl_3_·H_2_O)Br^7^, indicating successful introduction of Br^−^.


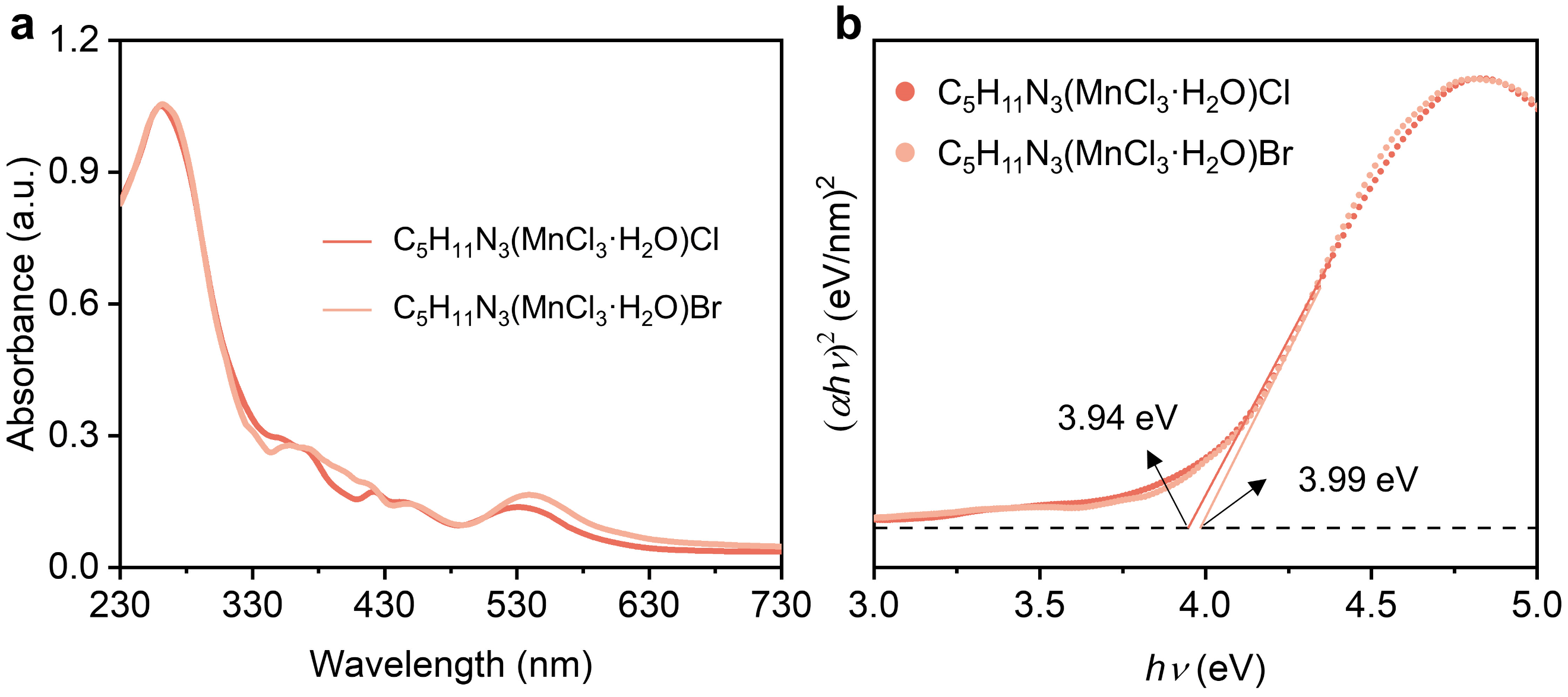


**Fig. S2** **a, b** Absorption spectra **(a)** and Tauc's plots **(b)** for C_5_H_11_N_3_(MnCl_3_·H_2_O)Br and C_5_H_11_N_3_(MnCl_3_·H_2_O)Cl.





**Fig. S3** Micrographs of C_5_H_11_N_3_(MnCl_3_·H_2_O)Cl (left) and C_5_H_11_N_3_(MnCl_3_·H_2_O)Br (right) under 365 nm and natural light.





**Fig. S4** CIE chromaticity diagram for the emission of C_5_H_11_N_3_(MnCl_3_·H_2_O)Cl and C_5_H_11_N_3_(MnCl_3_·H_2_O)Br.


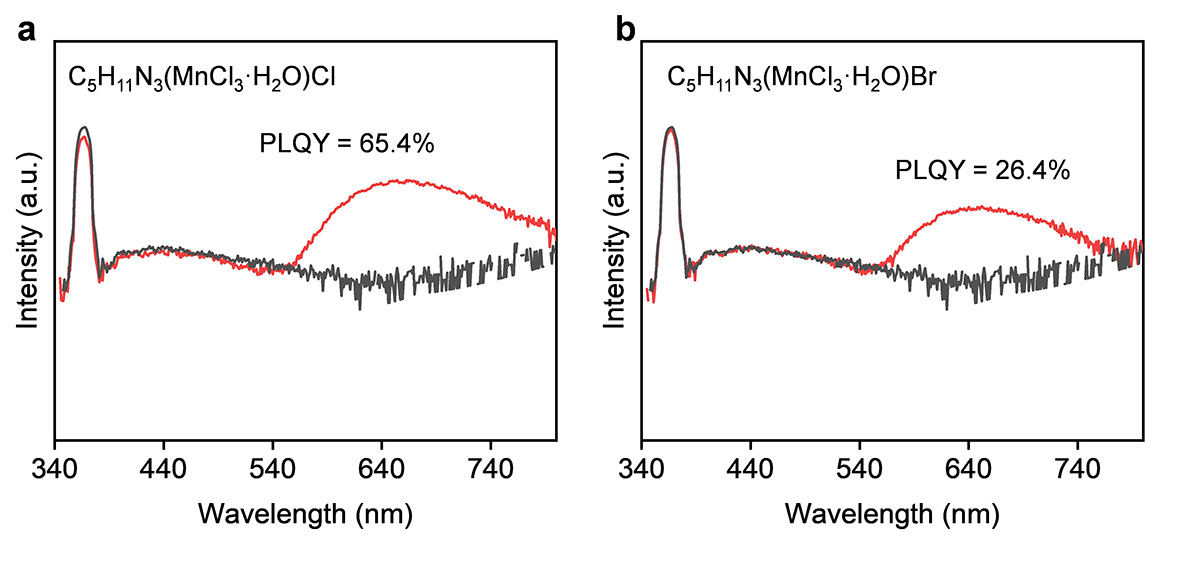


**Fig. S5 a, b** PLQY spectra of C_5_H_11_N_3_(MnCl_3_·H_2_O)Cl **(a)** and C_5_H_11_N_3_(MnCl_3_·H_2_O)Br **(b)**.


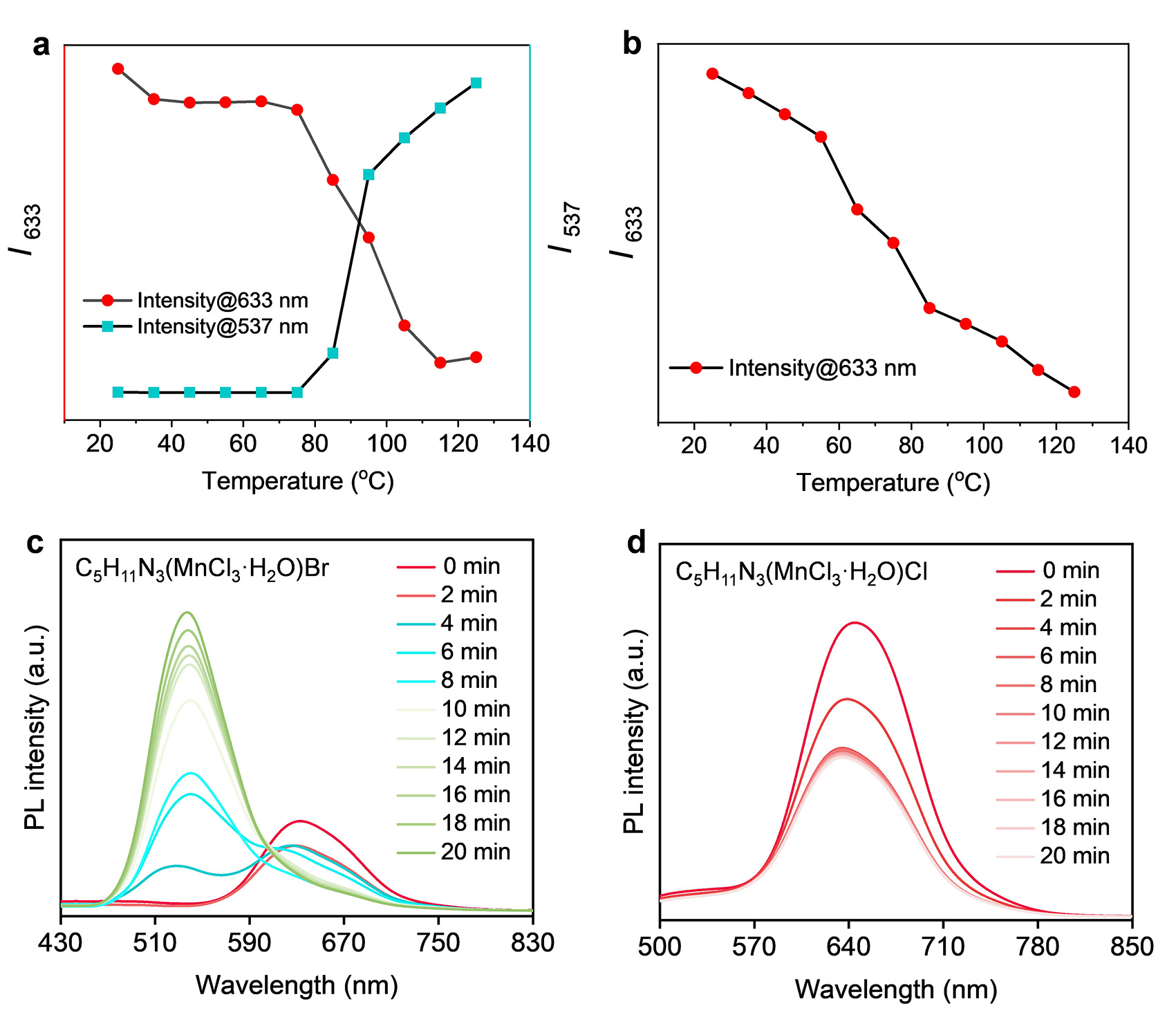


**Fig. S6** **a, b** Temperature-dependent PL intensity plots for C_5_H_11_N_3_(MnCl_3_·H_2_O)Br **(a)** and C_5_H_11_N_3_(MnCl_3_·H_2_O)Cl **(b)**. **c**, **d** PL spectra of C_5_H_11_N_3_(MnCl_3_·H_2_O)Br **(c)** and C_5_H_11_N_3_(MnCl_3_·H_2_O)Cl **(d)** under thermal stimulation (90 ℃) in air.

**Note**: As can be seen from the temperature-dependent PL intensity plots, the red PL band of C_5_H_11_N_3_(MnCl_3_·H_2_O)Br gradually decreases with the increasing temperature, while the green PL band gradually increases, indicating a thermally stimulated PL color change (**Fig. S6a**). In contrast, no green PL band appears throughout the heating process for C_5_H_11_N_3_(MnCl_3_·H_2_O)Cl, and only a gradually decreasing red PL band is observed (**Fig. S6b**). The red-to-green conversion in C_5_H_11_N_3_(MnCl_3_·H_2_O)Br can be accomplished in 10 min at 90 ℃, as shown in **Fig. S6c**. However, no spectral shift is observed in C_5_H_11_N_3_(MnCl_3_·H_2_O)Cl even when it has been heated at 90 ℃ for 20 min (**Fig. S6d**).


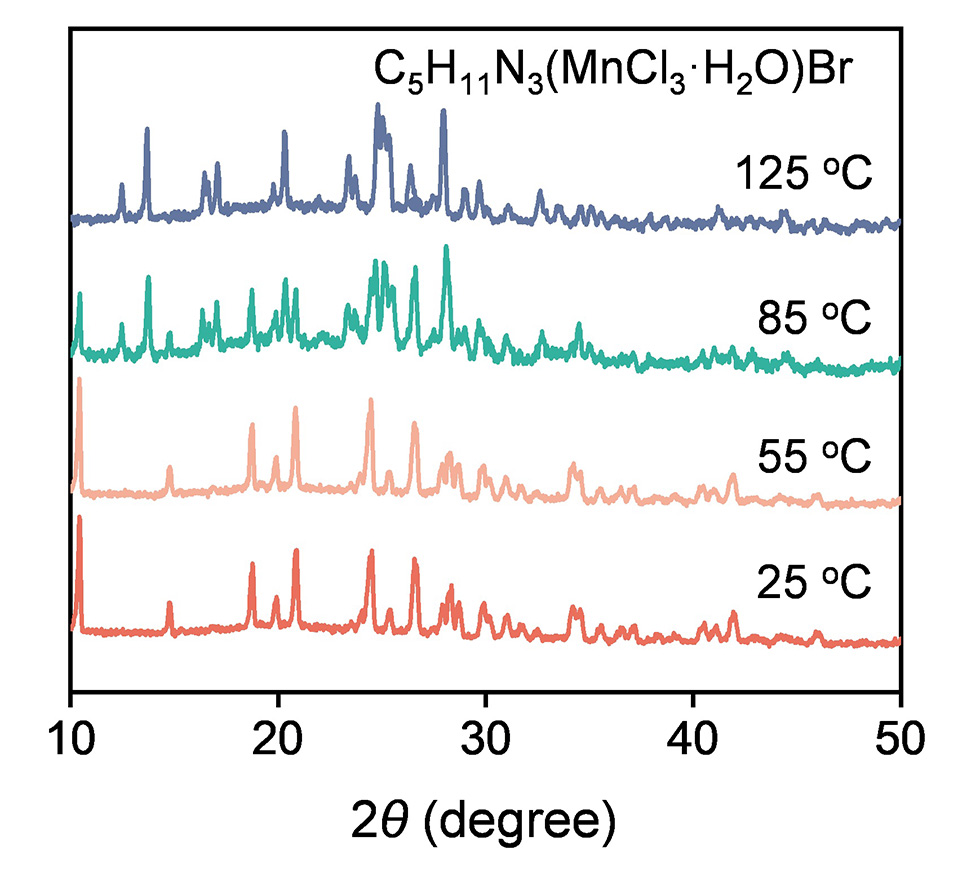


**Fig. S7** Temperature-dependent PXRD patterns of C_5_H_11_N_3_(MnCl_3_·H_2_O)Br in 25–125 ^o^C.





**Fig. S8** PL spectra of C_5_H_11_N_3_(MnCl_3_·H_2_O)Br during one heating-cooling cycle. Inset are the photographs after the corresponding step.





**Fig. S9** PXRD patterns of C_5_H_11_N_3_(MnCl_3_·H_2_O)Br after one heating-cooling cycle.





**Fig. S10** DSC curves of C_5_H_11_N_3_(MnCl_3_·H_2_O)Br and C_5_H_11_N_3_(MnCl_3_·H_2_O)Cl.


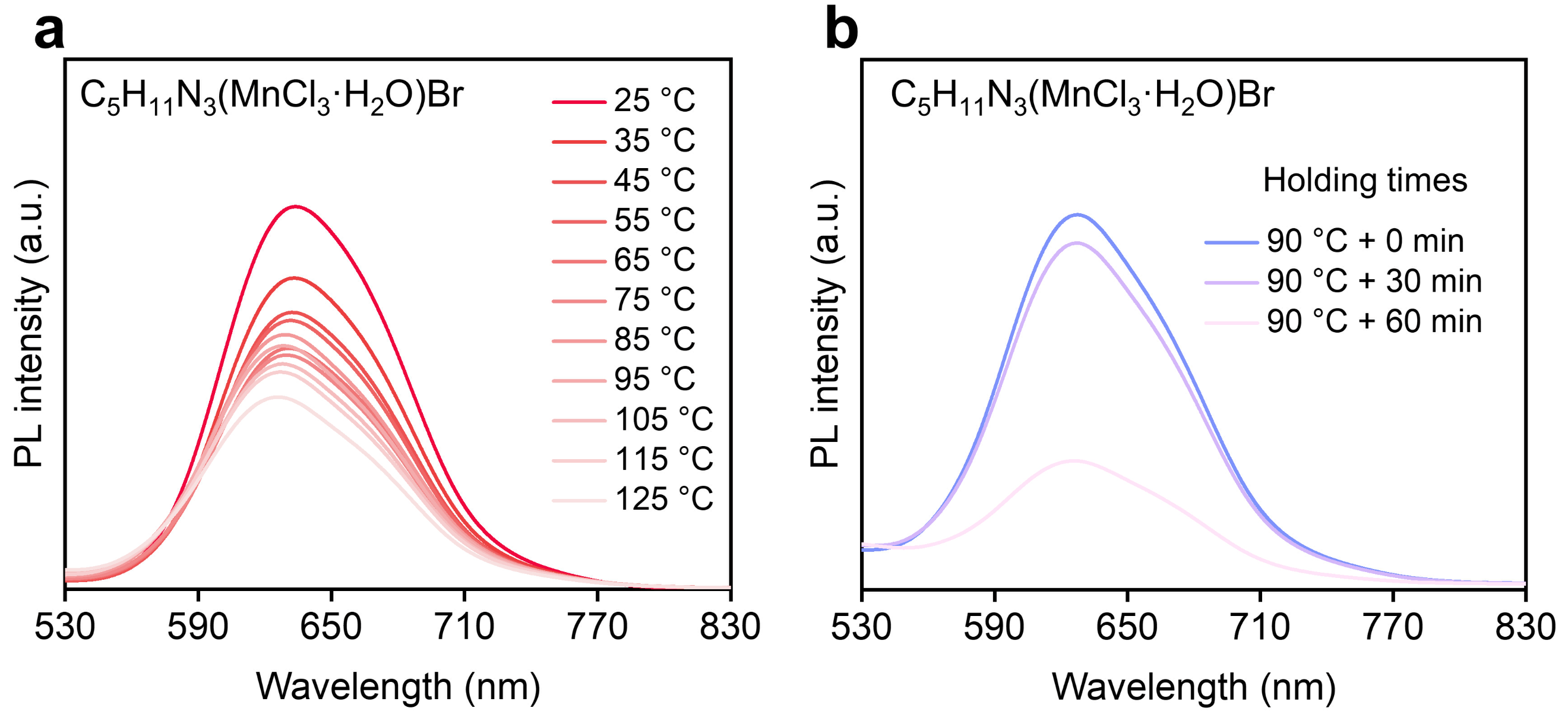


**Fig. S11 a** Temperature-dependent PL spectra of C_5_H_11_N_3_(MnCl_3_·H_2_O)Br. **b** PL spectra of C_5_H_11_N_3_(MnCl_3_·H_2_O)Br at 90 °C for different times. All the samples were sealed before the measurements to prevent H_2_O escape during the heating process.





**Fig. S12** Temperature-dependent PL spectra of C_5_H_11_N_3_(MnCl_3_·H_2_O)Br/PDMS film, with photographs on the right showing the luminescence color at different temperatures.


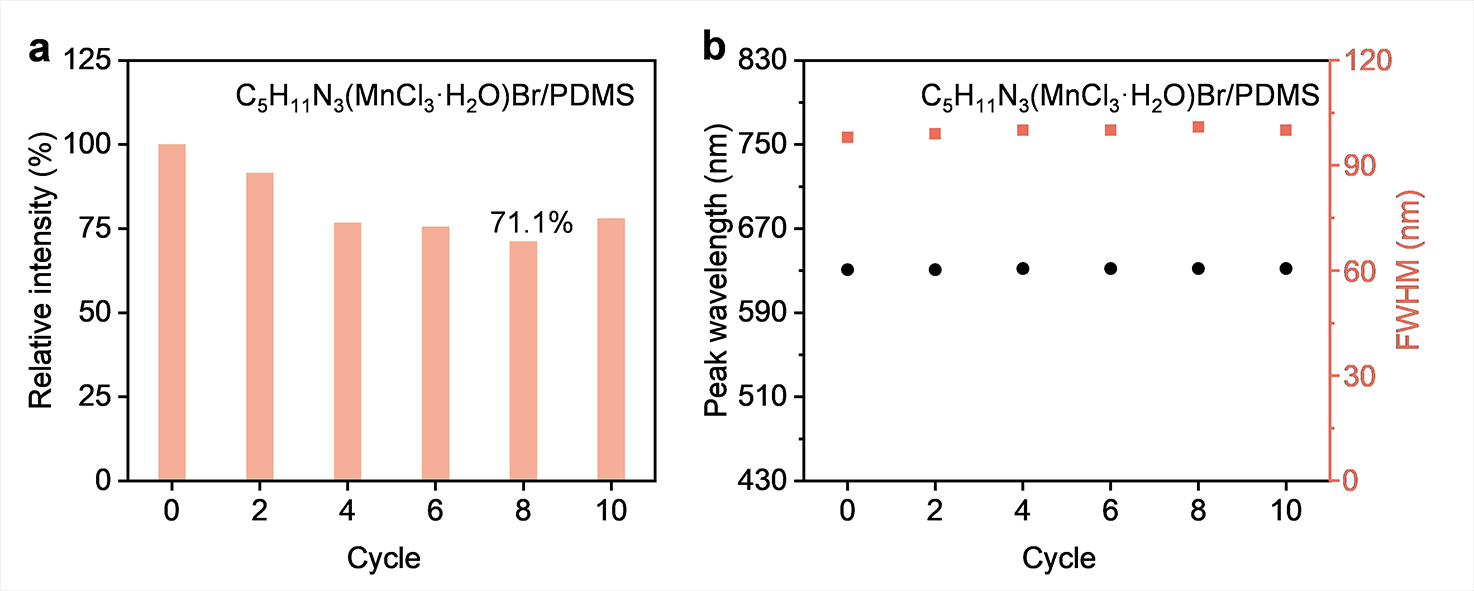


**Fig. S13 a,b** Relative PL intensity **(a)** and peak position and FWHM values **(b)** of C_5_H_11_N_3_(MnCl_3_·H_2_O)Br/PDMS within 10 cycles of heating and cooling.

**Note**: Temperature-dependent PL spectra and photographs of the C_5_H_11_N_3_(MnCl_3_·H_2_O)Br/PDMS film confirm that the introduction of PDMS does not affect the thermally-responsive PL performance of C_5_H_11_N_3_(MnCl_3_·H_2_O)Br. In addition, the film shows favorable reversibility and durability within 10 cycles of heating and cooling.

**Table S1**. Crystallographic data for C_5_H_11_N_3_(MnCl_3_·H_2_O)Br and C_5_H_11_N_3_(MnCl_3_·H_2_O)Cl.

| Empirical formula | C_5_H_11_N_3_(MnCl_3_·H_2_O)Cl | C_5_H_11_N_3_(MnCl_3_·H_2_O)Br |
| --- | --- | --- |
| CCDC | 2457548 | 2457549 |
| Formula weight/g·mol^−1^ | 326.81 | 372.38 |
| Temperature/K | 100.15 | 100.00 |
| Crystal system | tetragonal | tetragonal |
| Space group | $P\bar{4}2_{1}c$ | $P\bar{4}2_{1}c$ |
| *a*/Å | 18.47139(10) | 18.7077(3) |
| *b*/Å | 18.47139(10) | 18.7077(3) |
| *c*/Å | 7.17756(6) | 7.16122(17) |
| *α*/° | 90 | 90 |
| *β*/° | 90 | 90 |
| *γ*/° | 90 | 90 |
| Volume/Å^3^ | 2448.93(3) | 2506.27(9) |
| *Z* | 8 | 8 |
| *ρ*_calc_ (g/cm^3^) | 1.773 | 1.974 |
| *μ*/(mm^−1^) | 16.606 | 18.003 |
| *F* (000) | 1315.0 | 1464.0 |
| 2*θ* range (deg) | 9.576 to 146.578 | 9.456 to 145.732 |
| Reflections collected | 77206 | 9904 |
| Independent reflections | 2410 [*R*_int_ = 0.0751, *R*_sigma_ = 0.0186] | 2405 [*R*_int_ = 0.0495, *R*_sigma_ = 0.0452] |
| Goodness-of-fit on *F*^2^ | 1.087 | 1.055 |
| Final *R* indexes [I>=2*σ* (I)] | *R*_1_ = 0.0377, *wR*_2_ = 0.0802 | *R*_1_ = 0.0476, *wR*_2_ = 0.1269 |
| Final *R* indexes [all data] | *R*_1_ = 0.0380, *wR*_2_ = 0.0804 | *R*_1_ = 0.0509, *wR*_2_ = 0.1297 |
| Largest diff. peak/hole / e Å^−3^ | 0.48/−0.63 | 1.57/−1.22 |

**Table S2**. Bond lengths between central Mn^2+^ and coordinated ions in C_5_H_11_N_3_(MnCl_3_·H_2_O)Br and C_5_H_11_N_3_(MnCl_3_·H_2_O)Cl.

| Sample | C_5_H_11_N_3_(MnCl_3_·H_2_O)Cl | | | | | |
| --- | --- | --- | --- | --- | --- | --- |
| Mn-Cl/O | Mn-Cl (1) | Mn-Cl (2) | Mn-Cl (3) | Mn-Cl (4) | Mn-Cl (5) | Mn-O (1) |
| Bond length (Å) | 2.5741 | 2.4886 | 2.5102 | 2.5225 | 2.5059 | 2.2830 |
| Avg. value (Å) | 2.4807 | | | | | |
| Distortion index | 0.0267 | | | | | |
| Sample | C_5_H_11_N_3_(MnCl_3_·H_2_O)Br | | | | | |
| Mn-Cl/O | Mn-Cl (1) | Mn-Cl (2) | Mn-Cl (3) | Mn-Cl (4) | Mn-Cl (5) | Mn-O (1) |
| Bond length (Å) | 2.5010 | 2.6110 | 2.5665 | 2.5140 | 2.5600 | 2.2830 |
| Avg. value (Å) | 2.5059 | | | | | |
| Distortion index | 0.0303 | | | | | |

**Note:** The distortion index (DI) is quantified using the following formula^8^:

|  | $DI=\frac{1}{n}\sum_{i=1}^{n} \frac{\left\vert l_{i}-l_{av} \right\vert}{l_{av}}$ | (S1) |
| --- | --- | --- |

where *l_i_* is the distance between the central Mn and the coordinated halide ions or water molecule; *l*_aν_ is the average bond length.

**Table S3**. Fitted PL lifetime values for C_5_H_11_N_3_(MnCl_3_·H_2_O)Br and C_5_H_11_N_3_(MnCl_3_·H_2_O)Cl.

| Samples | *λ*_ex_ | *λ*_em_ | *τ*_1_/ms | *R*_2_ |
| --- | --- | --- | --- | --- |
| C_5_H_11_N_3_(MnCl_3_·H_2_O)Cl | 365 | 650 | 0.924 | 0.998 |
| C_5_H_11_N_3_(MnCl_3_·H_2_O)Br | 365 | 633 | 0.341 | 0.997 |

**Note**: The PL decay curves of C_5_H_11_N_3_(MnCl_3_·H_2_O)Cl and C_5_H_11_N_3_(MnCl_3_·H_2_O)Br can be fitted with the following single-exponential function:

|  | $I\left( t \right)=A_{1}\exp\left( \frac{-t}{\tau_{1}} \right)+y_{0}$ | (S2) |
| --- | --- | --- |

where *I*(*t*) represent the PL intensity, *τ*_1_ refers to decay lifetime, *A*_1_ and *y*_0_ are constants.

**Table S4**. Fitting results of Mn K-edge EXAFS spectra of C_5_H_11_N_3_(MnCl_3_·H_2_O)Br collected at 100 ℃.

| sample | Mn-Cl | | Mn-Br | | Δ*E*_0_(eV)^d^ | *R* factor |
| --- | --- | --- | --- | --- | --- | --- |
|  | *R*(Å) | CN | *R*(Å) | CN | 2.39 | 0.027 |
| C_5_H_11_N_3_(MnCl_3_·H_2_O)Br | 2.261 | 3.26 | 2.526 | 0.74 |  |  |

**Note**: *R* is the bond distance; CN is the coordination number; Δ*E*_0_ is an inner potential correction (the difference between the zero kinetic energy value of the sample and that of the theoretical model); the *R* factor is used to value the goodness of the fitting.

**3. References**

1. Kresse, G. & Furthmüller, J. Efficiency of ab-initio total energy calculations for metals and semiconductors using a plane-wave basis set. *Comput. Mater. Sci.* **6**, 15–50 (1996).
2. Perdew, J. P. et al. Generalized Gradient Approximation Made Simple. *Phys. Rev. Lett.* **77**, 3865–3868 (1996).
3. Blöchl, P. E. Projector augmented-wave method. *Phys. Rev. B* **50**, 17953–17979 (1994).
4. Kresse, G. & Joubert, D. From ultrasoft pseudopotentials to the projector augmented-wave method. *Phys. Rev. B* **59**, 1758–1775 (1999).
5. Anthony, A. M. et al. Zero- and one-dimensional lead-free perovskites for photoelectrochemical applications. *ACS Appl. Mater. Interfaces* **14**, 29735–29743 (2022).
6. Ren, J. et al. Highly luminescent tin-doped manganese halide perovskite nanocrystals for high-resolution patterning and light-emitting diodes. *Chem. Eng. J.* **488**, 150977 (2024).
7. Meng, Q. et al. Enhanced photoluminescence of all-inorganic manganese halide perovskite-analogue nanocrystals by lead ion incorporation. *J. Phys. Chem. Lett.* **12**, 10204–10211 (2021).
8. Panda, D. P. et al. Negative thermal quenching and self-trapped exciton emission in (*R*/*S*-C_3_H_10_ON)MnCl_3_. *Chem. Mater.* **36**, 5698–5708 (2024).
